# Preparation of Zeolite X by the Aluminum Residue From Coal Fly Ash for the Adsorption of Volatile Organic Compounds

**DOI:** 10.3389/fchem.2019.00341

**Published:** 2019-05-14

**Authors:** Tao Zhu, Xing Zhang, Yiwei Han, Tongshen Liu, Baodong Wang, Zhonghua Zhang

**Affiliations:** ^1^Institute of Atmospheric Environmental Management and Pollution Control, China University of Mining and Technology (Beijing), Beijing, China; ^2^State Environmental Protection Key Laboratory of Odor Pollution Control, Tianjin, China; ^3^Beijing Municipal Research Institute of Environmental Protection, Beijing, China; ^4^National Institute of Clean-and-Low-Carbon Energy, Beijing, China

**Keywords:** coal fly ash, aluminum residue, zeolite X, activated carbon, adsorption performance, volatile organic compounds

## Abstract

In China, coal fly ash is a large-scale solid waste generated by power plants. The high value utilization of coal fly ash has always been a hot research issue in China for these years. In this paper, the synthesis of zeolite X using aluminum residue from coal fly ash can not only realize the resource utilization of waste, but also achieve the effect of energy saving and emission reduction. Zeolite X prepared by hydrothermal synthesis method have been found to have higher purity and better crystallinity by chemical composition analysis. By comparing and analyzing the adsorption performance of zeolite X and activated carbon on volatile organic compounds, it is found that the adsorption capacity of zeolite X is higher than that of activated carbon, and it has stronger stability. This indicates that the zeolite X synthesized by this environmentally friendly and economical method has a good application prospect in adsorbing volatile organic compounds.

## Introduction

According to the definition of the World Health Organization, volatile organic compounds (VOCs) usually refer to organic compounds having boiling points within the range of 50~260°C (Kuo et al., 2014). VOCs are widely distributed in air, water, soil and other environmental media and have high risk biological toxicity properties, which pose significant threat to the human health (Ramos et al., 2010). VOCs are important atmospheric pollutants, which are precursors and participants of PM_2.5_ that can not only react with nitrogen oxides to form photochemical smog, but also react with hydroxyl and other strong oxidants in the atmosphere to form secondary organic aerosols (Wei et al., 2014a). In recent years, the multiple environmental effects on regional atmospheric are caused by VOCs, which has become one of the major problems for human survival and development (Wei et al., 2014b).

The adsorption method is widely used in gas purification and it has the advantages of mature process, simple operation, high purification efficiency and low energy consumption compared to other methods (Rioland et al., 2017). Therefore, the adsorption method is often used to deal with industrial organic gas with high air volume and low concentration. The selection of adsorbent is the key to the purification of VOCs by adsorption (Son et al., 2016). The ideal adsorbent should be characterized by large specific surface area, high adsorption capacity, reversible adsorption, strong hydrophobicity, high thermal stability and easy regeneration (Long et al., 2011). At present, activated carbon is widely used as adsorbent in gas purification. Although the adsorption capacity of activated carbon is strong, there are flammable and hydrophilic in the adsorption process which is difficult to absorb the complex and changeable industrial organic gas. Pen et al. studied the performance of activated carbon in the adsorption of VOCs and found that the activated carbon has problems such as poor stability and pore blockage, which strongly hinders the adsorption of VOCs (Chiang et al., 2001).

Zeolite X is a kind of nanocrystalline material with skeleton structure, which has large specific surface area and pore volume due to its special crystal structure and uniform pore channels. Therefore, zeolite X is characterized by strong adsorption and selection capabilities for VOCs. In addition, zeolite X shows excellent hydrophobic and hydrothermal stability, which effectively reduces the competitive adsorption of water molecules, so zeolite X is widely used to adsorb VOCs (Ling et al., 2016; Brihi et al., 2018). However, the reserve of zeolite X is scarce in nature and the adsorption performance usually cannot meet the requirements of industrial applications. Zeolite X is generally synthesized by aluminum hydroxide, sodium silicate and sodium aluminate in the industrial production, which consumes a lot of energy and may cause severe secondary pollution. Coal fly ash contains various valuable elements such as silicon and aluminum that are required for the preparation of zeolite X. At present, using coal fly ash to synthesize zeolite X has attracted wide attention from numerous scholars and it has shown great application in the fields of gas purification and sewage treatment. Zhang et al. used different concentrations of hydrochloride and sodium hydroxide solution to modify coal fly ash for preparing zeolite. He found that the benzene adsorption capacity of zeolite can reach 151 mg/g, which is very close to or even exceeds commercial activated carbon (Zhang et al., 2012). Although many scholars have conducted in-depth research on the synthesis of zeolite using coal fly ash, the prepared zeolite X exhibits excellent adsorption performance and meet the requirements of industrial applications. However, a large number of residues are difficult to be recycled and various mineral resources contained in the coal fly ash cannot be fully utilized in the preparation process. This study differs from others is that the zeolite X is obtained from the aluminum residue. So the aluminum residue is resourced and the comprehensive utilization efficiency of coal fly ash is improved. It saves not only a lot of chemical raw materials, but also preparation cost (Brunchi et al., 2012; Zhang et al., 2012; Huang et al., 2014; Yuan et al., 2015; Rioland et al., 2017).

## Materials and Methods

### Preparation of Zeolite X

The coal fly ash used in the present study was obtained from an electric power plant in Inner Mongolia, China. Concentrated hydrochloride and sodium hydroxide were bought from the Sinopharm Chemical Reagent Co., Ltd. The coal fly ash was dried at 150°C for 12 h before the experiments and then it was leached with hydrochloride to form aluminum chloride. The remaining aluminum residues were mainly non-metallic inorganic mineral. The aluminum residue can be used as high-quality aluminosilicate material due to its small particle size and high activity.

**Figure 1:**
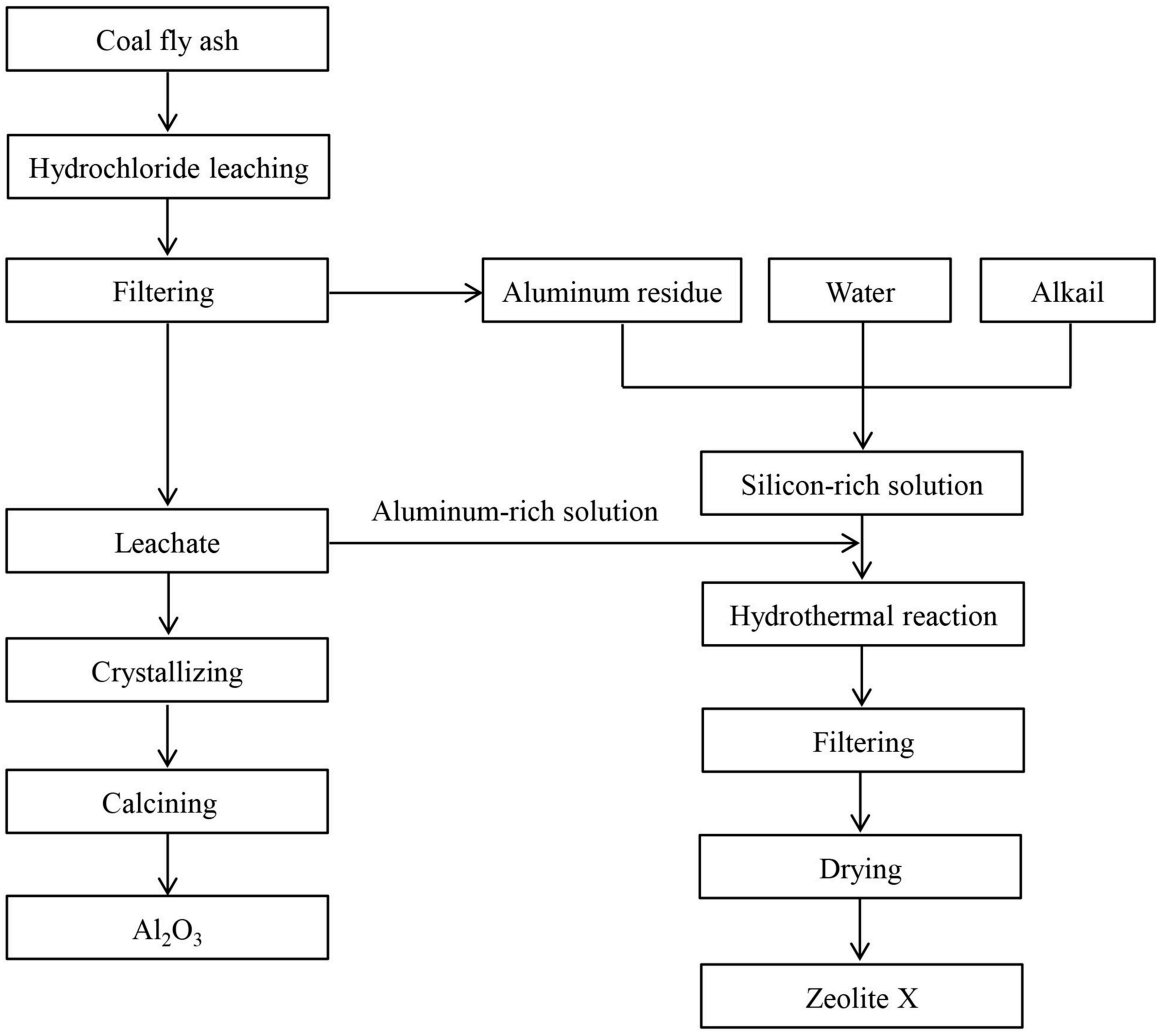
Process diagram for synthesizing zeolite X by aluminum residue.

The hydrothermal synthesis method with mild reaction conditions was used to synthesize zeolite X. The main chemical components of zeolite X were derived from the aluminum-rich solution and the silicon-rich solution in the synthesis process. The aluminum-rich solution was taken from the filtrate during the aluminum extraction process. The silicon-rich solution was obtained by filtration and the alkalinity of the silicon-rich solution was adjusted by introducing different volumes of carbon dioxide. The aluminum residue, sodium hydroxide and water were stirred and mixed at the mass ratio of 100:60:400 to obtain gelatinous mother liquor. The mixture was placed in the vessel and hydrothermal reaction was carried out at the temperature of 95°C for 20 h. The reaction product was filtered and washed until the pH of the washing liquid was 7. The product of zeolite X was obtained after drying. The main instruments used in the study were the 2L hydrothermal vessel and the CS501 constant temperature circulating tank (Figure 1).

**Figure 2:**
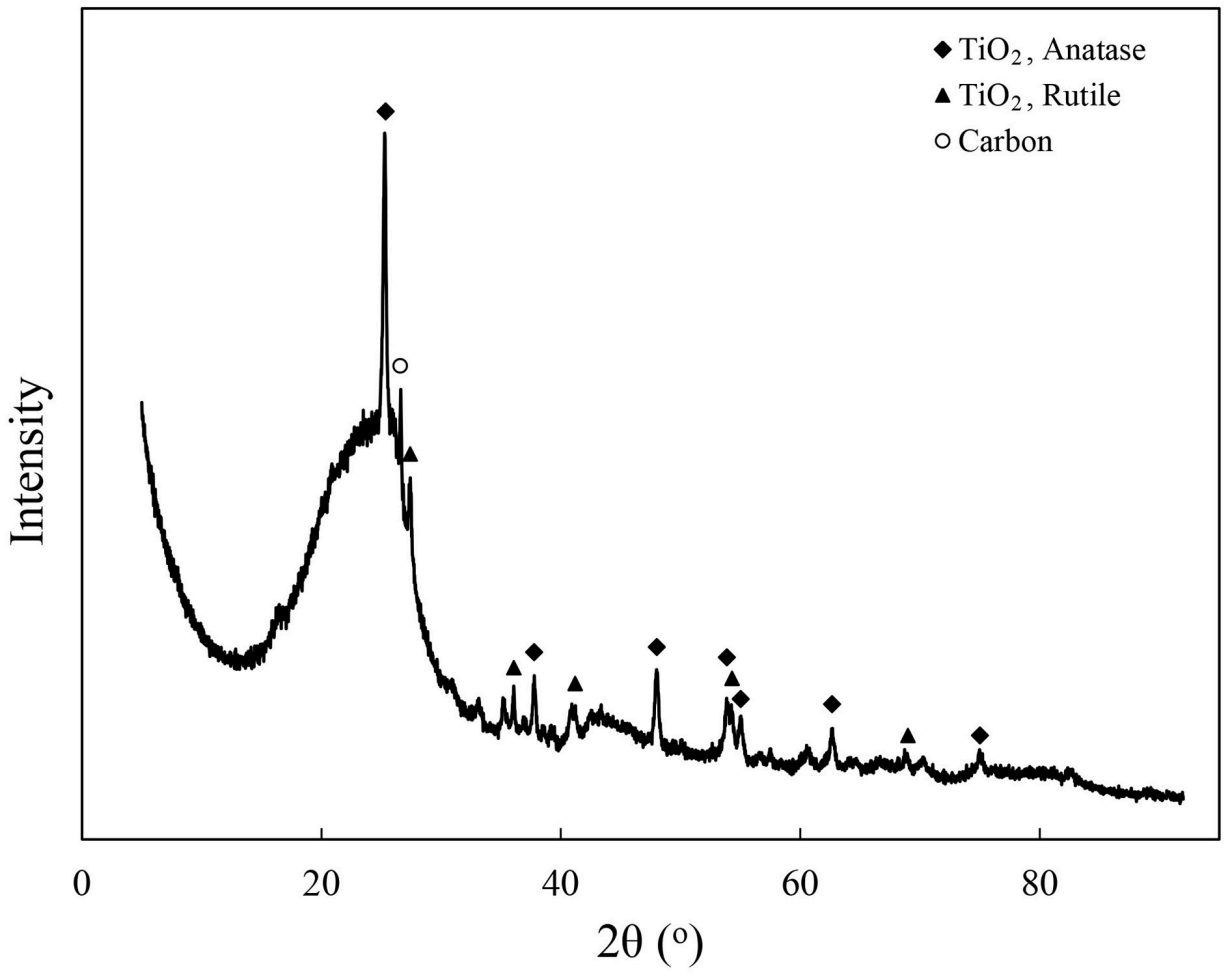
XRD diffraction pattern of aluminum residue.

The coal fly ash and the aluminum residue are rich in silicon and aluminum. The total contents of silica and alumina are 89.4 and 92.1%, respectively (Table 1). From the XRD diffraction pattern of the aluminum residue, it is known that most of the aluminum residue is amorphous phase material (Figure 2). The XRD pattern shows that the obvious peaks is between 20 and 30°. Its main phase is amorphous silica, mullite and quartz. In addition, a small amount of titanium dioxide minerals and some unburned carbon residue are present.

**Table 1:** Chemical analysis of coal fly ash and aluminum residue.

**Chemical composition**	**SiO_**2**_**	**Al_**2**_O_**3**_**	**K_**2**_O**	**CaO**	**TiO_**2**_**	**Fe_**2**_O_**3**_**	**SrO**
Coal fly ash/%	35.0	54.4	0.404	3.77	2.43	1.99	0.12
Aluminum residue/%	78.7	13.4	0.163	0.37	5.2	0.445	0.03

**Figure 3:**
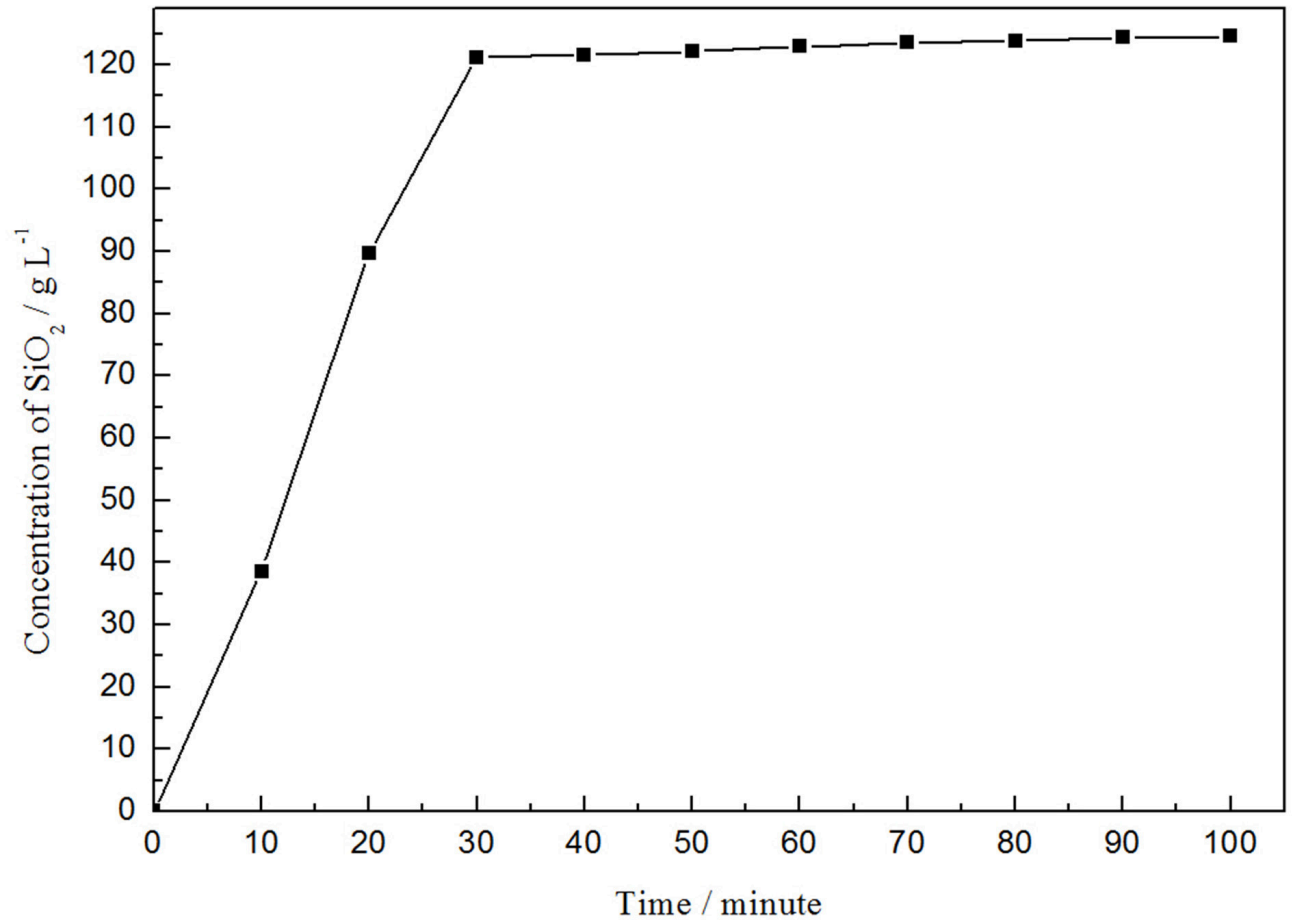
Variation of silica concentration with reaction time.

It can be seen that the reaction of aluminum residue and alkali is very rapid (Figure 3). The concentration of silicon dioxide is substantially stabilized at the maximum when the reaction takes place for 30 min. Thereafter, the concentration of silica changes slightly as the reaction time increases. Therefore, the optimum reaction time for desiliconization of alkali-washed aluminum residue is 30 min and the concentration of silicon dioxide in the silicon-rich solution is between 120 and 125 g•L^−1^.

### Characterization

The phase analysis of zeolite X was carried out on the Rigaku RINT-2000 X-ray diffraction (XRD) with the operating voltage of 40 kV, electric current of 100 mA, scanning speed of 0.02°/s and the scanning range of 5~80°. The microstructure was observed by the NOVA SEM 450 scanning electron microscope (SEM) with the operating voltage of 5.0 kV. The specific surface area and pore volume of the synthesized zeolite X were characterized by low temperature N_2_ adsorption-desorption method used the Micrometrics ASAP2020C type adsorber. The pretreatment was carried out before the adsorption measurement and the sample was desorbed at 250°C for more than 2 h. The specific surface area of the sample were calculated by the BET method. The pore volume was calculated as the amount of adsorption when the adsorbate was at the relative pressure P/P_0_ of 0.99. The pore size distribution was measured by the BJH method and based on the desorption branch of the adsorption-desorption isotherm.

### Adsorption Experiment of VOCs

**Figure 4:**
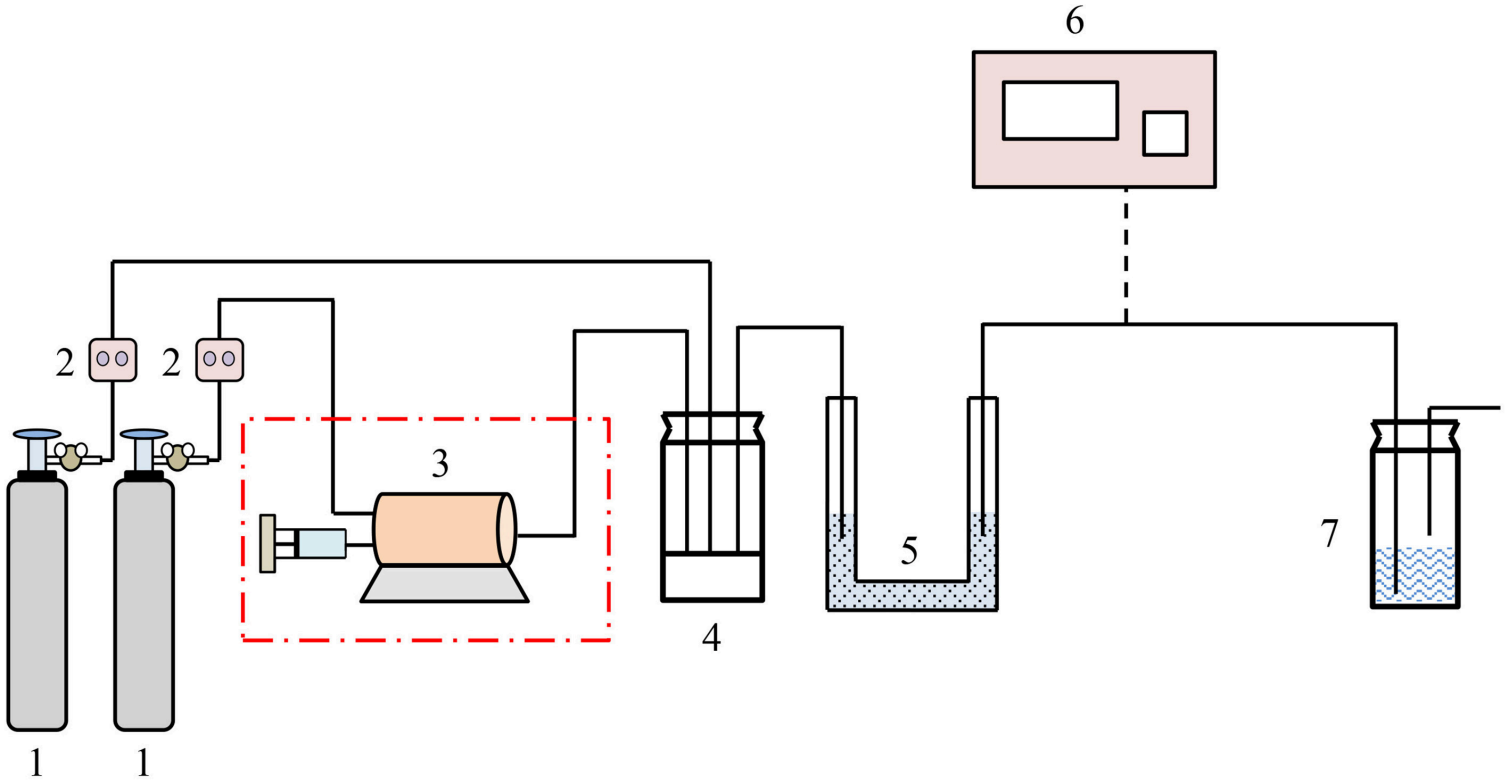
VOCs adsorption experimental apparatus.

The experimental device was consisted of gas distribution system, adsorption system and detection system (Figure 4). The gas distribution system used nitrogen as carrier gas. One part of nitrogen entered the gas chamber through trace syringe to carry out VOCs vapor and the other part acted as dilution gas. The flow of gas was regulated by mass flow meter. The concentration of VOCs was controlled by regulating the flow of nitrogen in both parts. The U-shaped tube was used as adsorbent bed. The zeolite X was desorbed by nitrogen for 3 h to remove water vapor and small amount of organic compounds at 400°C. The zeolite X was shaped into 20~30 mesh granules after screening and then ten grams of samples were loaded into the adsorption bed. Panna A91Gas Chromatography—Mass Spectrometer (GC-MS) was used to analyze the VOCs. The instrument column was HP-5 MS and it was programmed to raise the temperature. The initial temperature of 50°C was maintained for 1 min and then raised to 170°C at the heating rate of 15°C▪min^−1^. The temperature of front inlet and gasification chamber were 170 and 280°C respectively. The helium was used as carrier gas at the flow rate of 1 mL▪min^−1^. The electron ionization EI source was 70 eV and the temperature of ion source was 230°C.

1. Gas cylinder 2. Mass flow meter 3. VOCs generator 4. Mixing bottle 5. Adsorbent bed 6. GC-MS 7. Tail gas absorption

The adsorption volume of VOCs is calculated by integrating the adsorption curve. The mathematical expression is as follows:

(1) $$\begin{array}{r} q=\frac{\text{F}C_{0}10^{-6}}{\text{W}}\left[t_{s}-\int_{0}^{t_{s}}\frac{C_{i}}{C_{0}}dt\right] \end{array}$$

In this equation, q represents the saturated adsorption amount, in mg▪g^−1^. F stands for the flow rate of gas, in mL▪min^−1^. *C*_0_ stands for the concentration of VOCs at the entrance, in mg▪m^−3^, *C*_*i*_ stands for the concentration of VOCs at the exit of i minute, in mg▪m^−3^. W is the quality of the adsorbent, in g. *t*_*s*_ is the adsorption saturation time, in minutes.

## Results and Discussion

### Analysis of Chemical Phase

**Figure 5:**
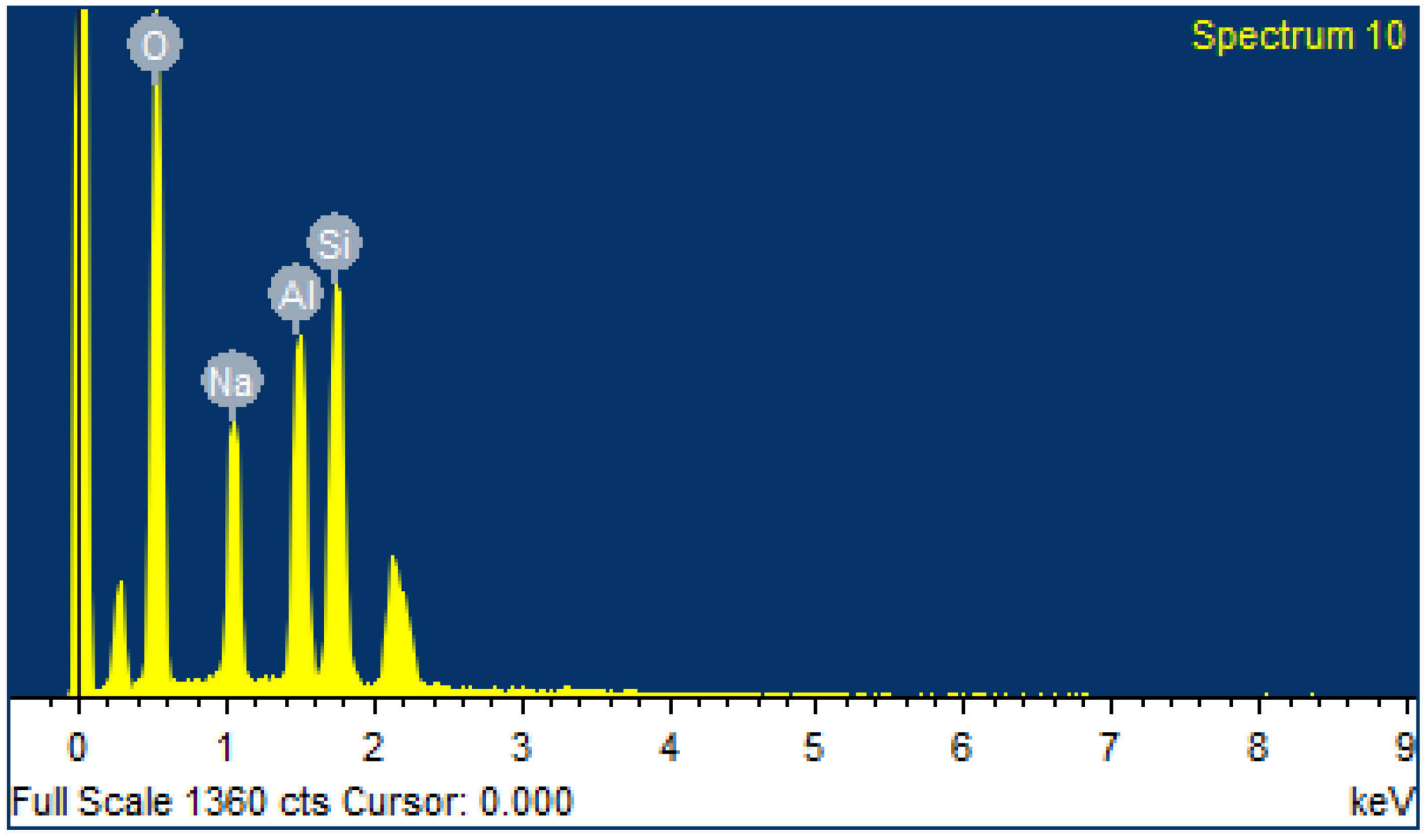
EDS analysis of zeolite X samples.

From the XRF analysis of sample, it can be seen that the mass of Na_2_O, Al_2_O_3_, and SiO_2_ in the zeolite X reaches 99.4% (Table 2). The impurities include S, K, and Fe, which accounts for 0.586%. It indicates that the prepared zeolite X sample is of high purity. Figure 5 and Table 3 show the EDS and elemental analysis of the zeolite X samples, respectively. Since EDS is primarily directed to the specific space of zeolite, it is commonly used to characterize individual particles or local samples. The analysis shows that the molar ratio of Si/Al in the zeolite X is about 1.33 and the molar ratio of SiO_2_/Al_2_O_3_ is about 2.66. The chemical composition of the synthesized zeolite is Na_2_O•Al_2_O_3_•(2.8±0.2)SiO_2_•(6~7)H_2_O.

**Table 2:** XRF analysis of zeolite X samples.

**Chemical composition**	**Na_**2**_O**	**Al_**2**_O_**3**_**	**SiO_**2**_**	**SO_**3**_**	**K_**2**_O**	**Fe_**2**_O_**3**_**
Weight/%	18.2	32.3	48.9	0.114	0.413	0.0589

**Table 3:** Elemental analysis of zeolite X samples.

**Element**	**O**	**Na**	**Al**	**Si**
Weight/%	53.91	12.74	14	19.34
Atomic/%	65.66	10.8	10.11	13.42

**Figure 6:**
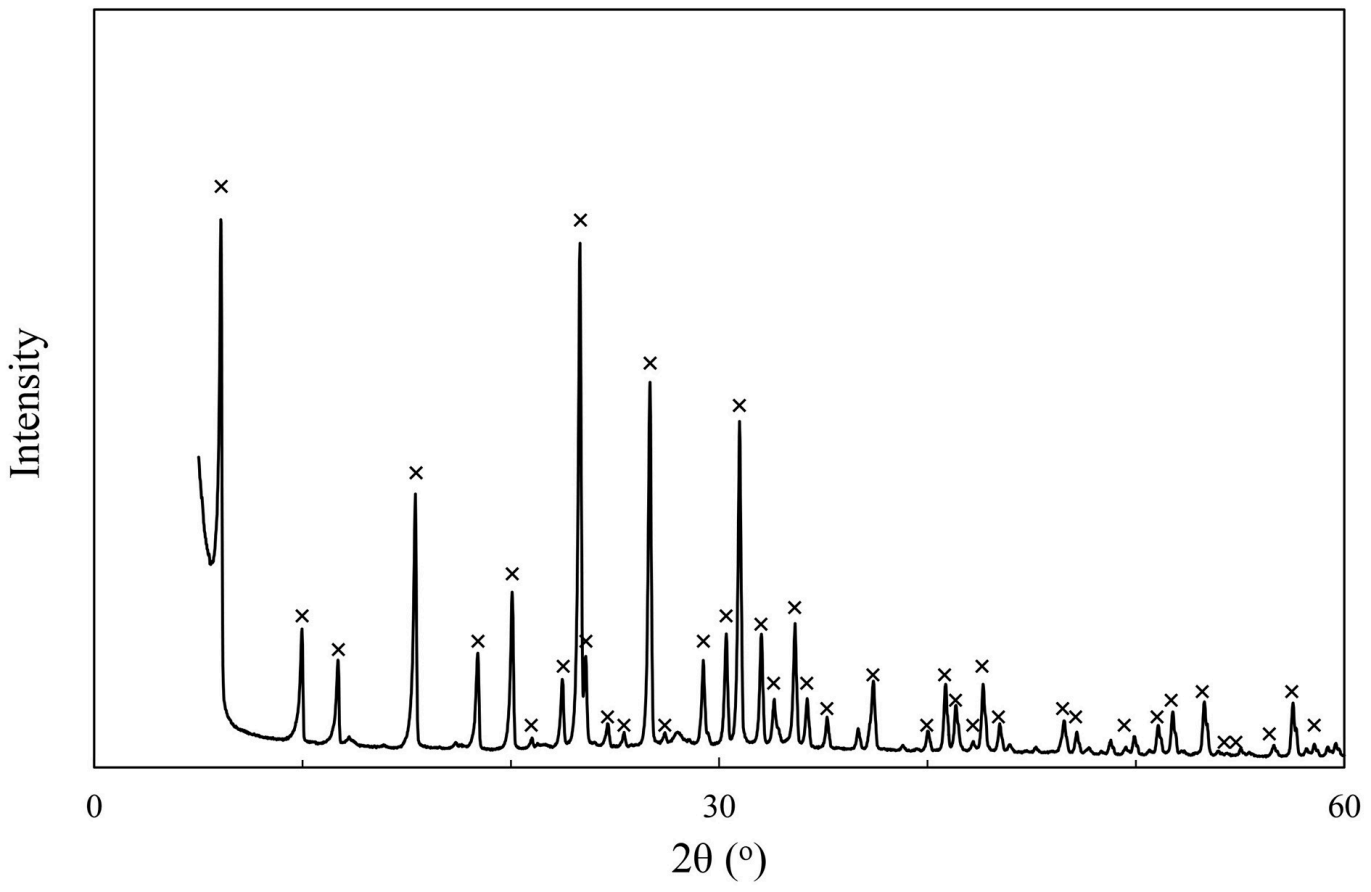
XRD diffraction pattern of zeolite X samples.

The XRD diffraction pattern of the zeolite X sample is shown in Figure 6. As can be seen from the figure, there is almost no amorphous peak in the XRD diffraction pattern of the zeolite X, which indicates that the zeolite X is of good crystallinity and no other crystals are formed.

### Analysis of Structure and Microstructure

**Figure 7:**
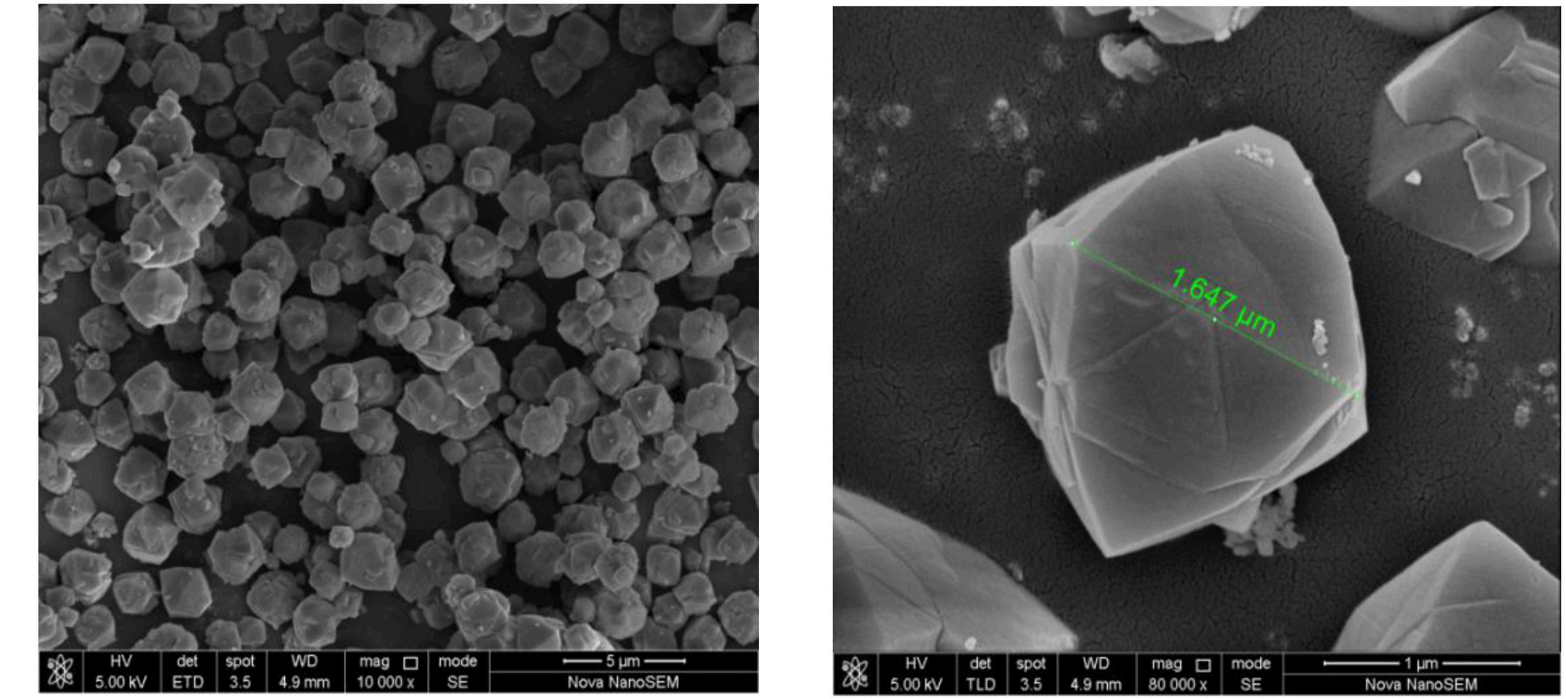
SEM image of zeolite X samples.

The specific surface area and pore structure are important indicators to evaluate zeolite. The BET specific surface area of the synthesized zeolite X is 990.3 m^2^/g and the pore diameter is about 1.6 nm. The microscopic morphology can reflect the crystallinity of the material. The SEM topography of the zeolite X sample is shown in Figure 7. It can be seen that the crystal form of the zeolite X is mainly octahedral structure. The particle size of zeolite X is between 1 and 3 μm which indicates that the particle size distribution is uniform. The surface of the partial particle unit cell is rough and has some defects. It may be that the aluminosilicate ions are not completely dissolved in water and some of the ions are still adsorbed on the surface of the particles during the hydrothermal reaction. It also may be due to the presence of Ca, K impurities in the mother liquor.

### Adsorption Results of VOCs

The penetration point is set as the VOCs concentration of outlet gas reaches 5% of the inlet gas. The time from the start of adsorption to the penetration point is the penetration time. The capacity of adsorption during the penetration time is penetration adsorption capacity. The adsorption saturation is set as the VOCs concentration of outlet gas reaches 100% of the inlet gas. The time from the start of adsorption to the adsorption saturation is the saturation time. The capacity of adsorption during the saturation time is the saturated adsorption capacity. The physical parameters of VOCs are shown in Table 4. The adsorption of isopropyl alcohol, benzene and cyclohexane was tested by the zeolite X and activated carbon and then comparing the adsorption properties of two adsorbents. The activated carbon was bought from Jiangsu Zhu Hai Environmental Technology Co., Ltd, and the BET specific surface area was 1020.8 m^2^/g and the pore diameter was 2.7 nm.

**Table 4:** Physical parameters of VOCs.

**VOCs**	**Relative molecular mass/g•mol^**−1**^**	**Density/g•mL^**−1**^**	**Boiling Point/^**°**^C**	**Tension/kPa**	**Molecular diameter/nm**
Cyclohexane	84	0.78	80.7	10.34	0.61
Benzene	78	0.88	80.1	10.03	0.53
Isopropanol	60	0.79	82.4	4.400	0.47

**Figure 8:**
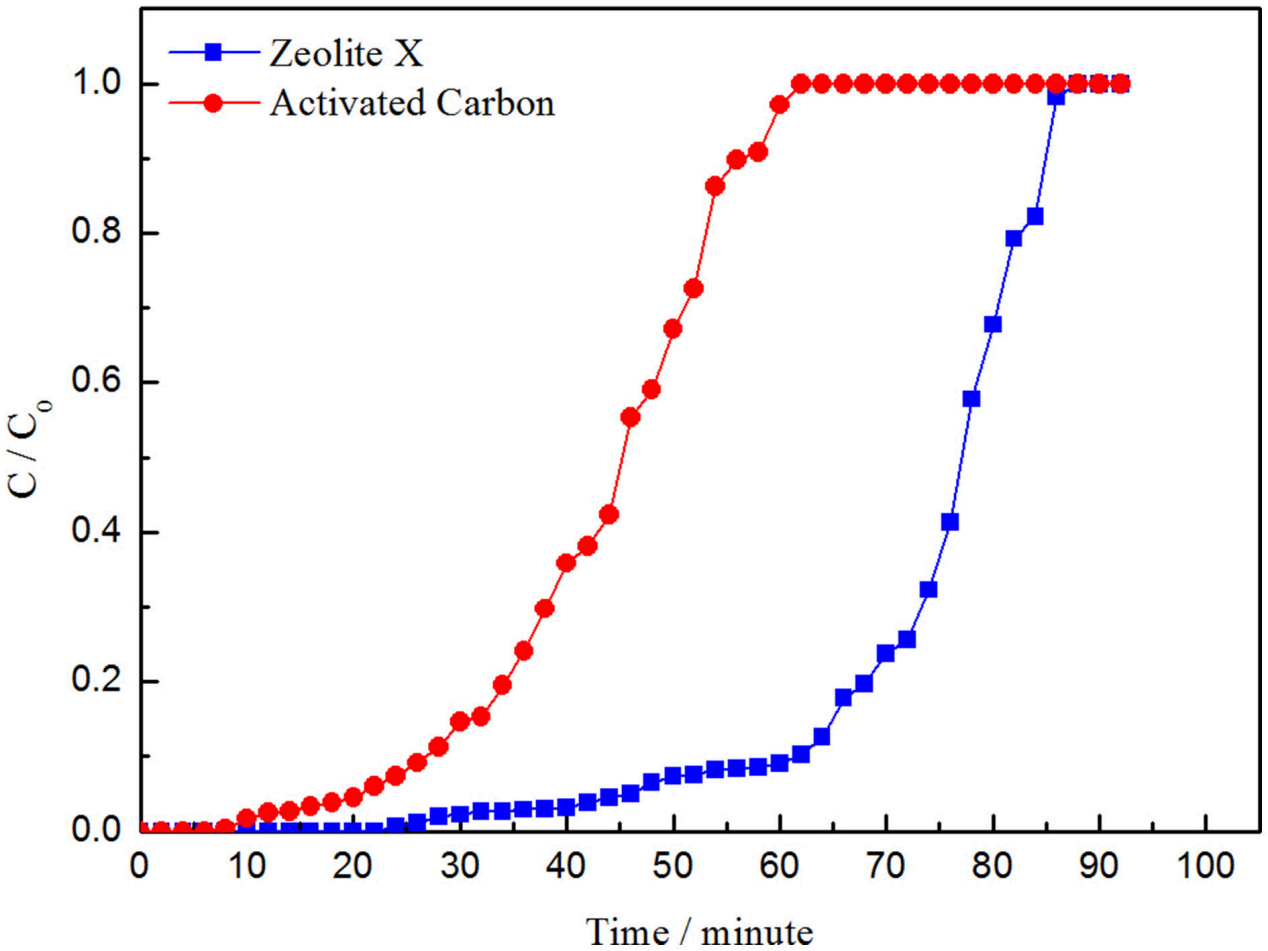
Adsorption curves of cyclohexane by zeolite X and activated carbon.

**Figure 9:**
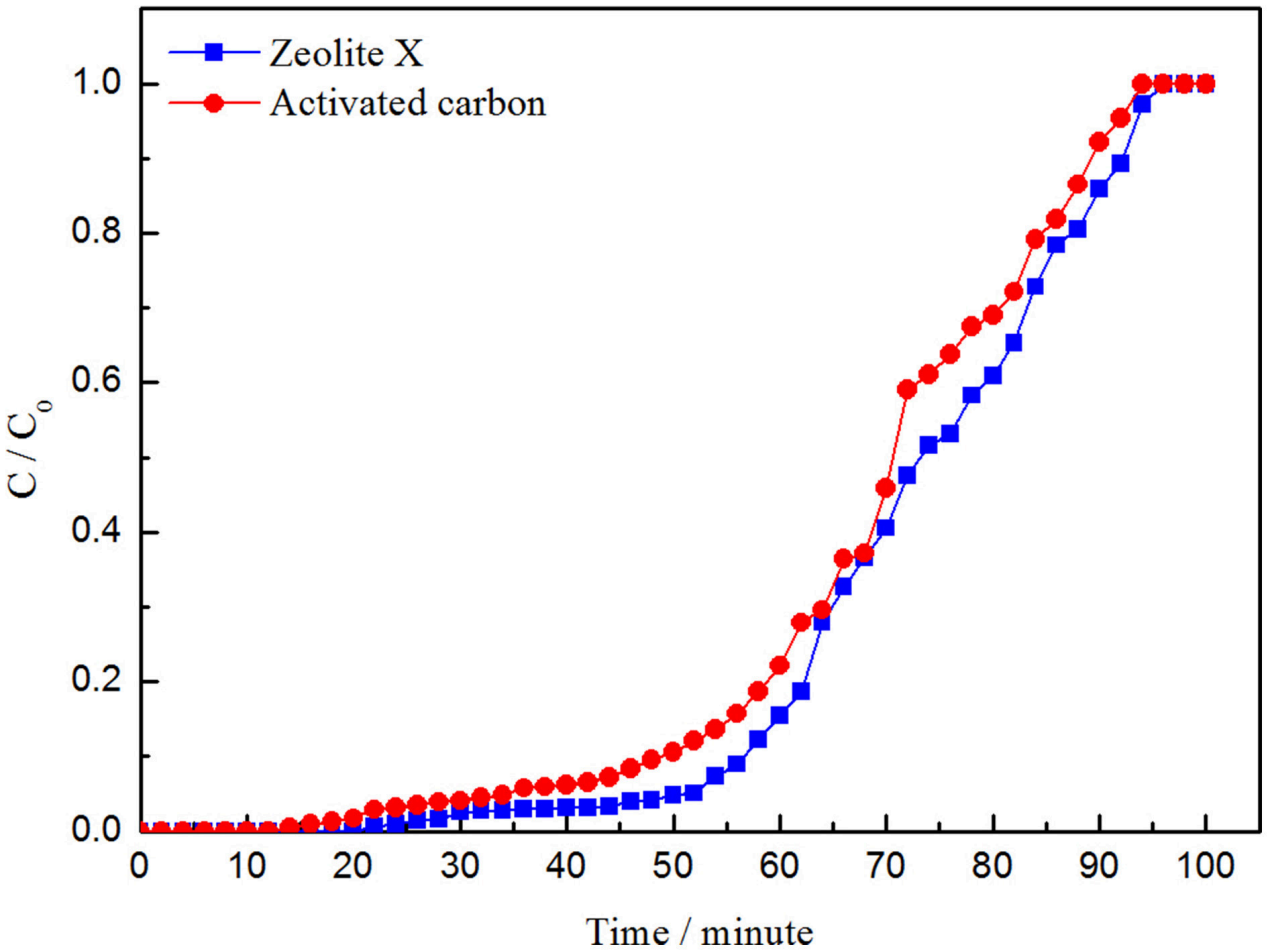
Adsorption curves of benzene by zeolite X and activated carbon.

**Figure 10:**
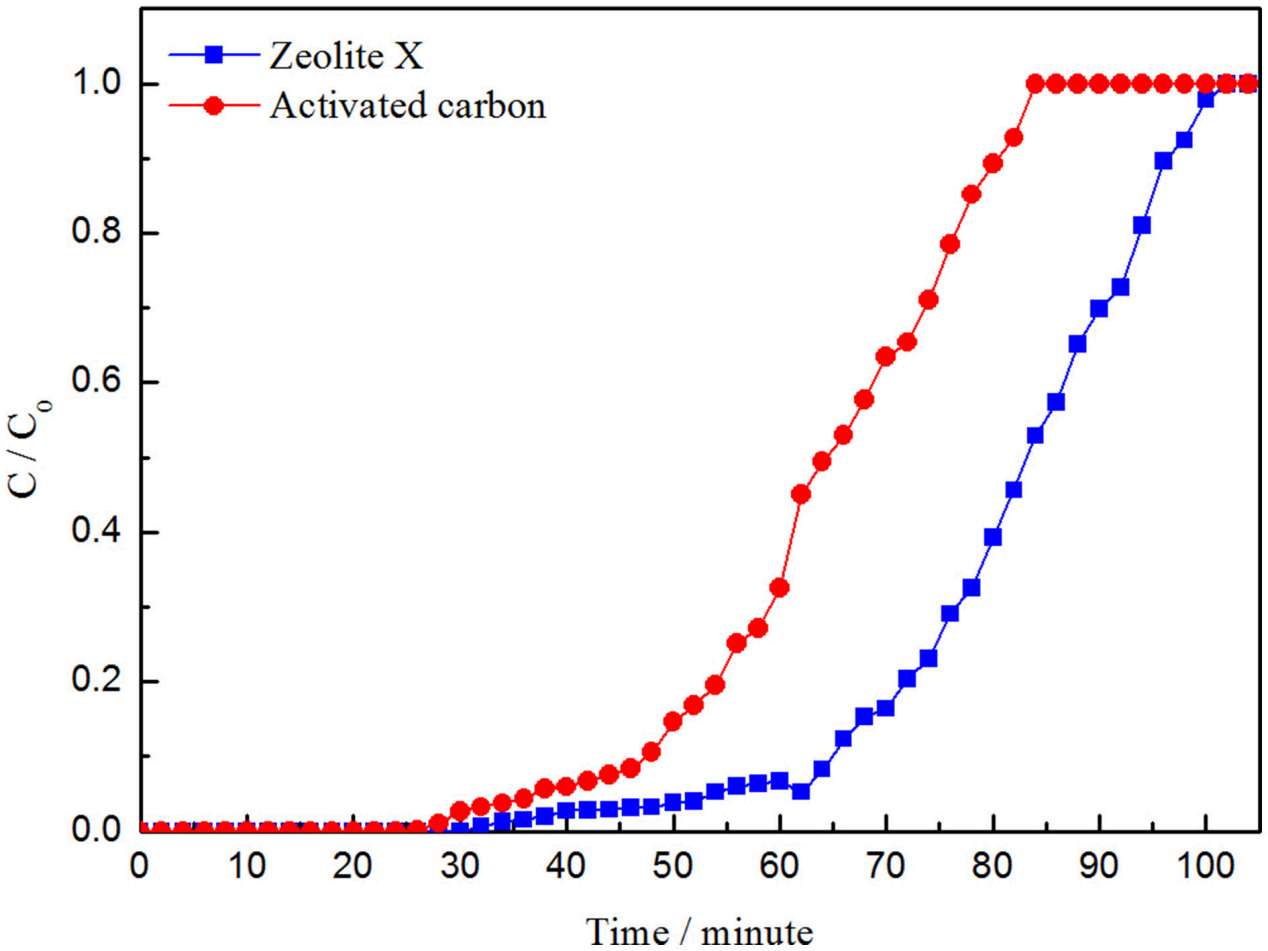
Adsorption curves of isopropanol by zeolite X and activated carbon.

As is shown in the picture, the adsorption curve of zeolite X lags behind that of activated carbon and the adsorption capacity of zeolite X on VOCs is larger than activated carbon, which indicates that zeolite X has better adsorption performance than activated carbon. The adsorption capacity of isopropanol is higher than that of benzene and cyclohexane, whether zeolite X or activated carbon (Figures 8–10; Tables 5, 6). The adsorption performance of the adsorbent is closely related to the polarity of the molecules. The polarity of isopropanol is stronger than that of benzene and cyclohexane. In addition, the unsaturation of molecules is also an important factor to determine the adsorption performance of adsorbent. The greater unsaturation of the molecules and the stronger adsorption capacity of the adsorbent.

**Table 5:** Adsorption performance of VOCs by zeolite X.

**VOCs**	**Penetration time/minute**	**Penetration adsorption capacity/mg•g^**−1**^**	**Saturated time/minute**	**Saturated adsorption capacity/mg•g^**−1**^**
Cyclohexane	46	16	88	117
Benzene	52	23	96	136
Isopropanol	54	27	102	141

**Table 6:** Adsorption performance of VOCs by activated carbon.

**VOCs**	**Penetration time/minute**	**Penetration adsorption capacity/mg•g^**−1**^**	**Saturated time/minute**	**Saturated adsorption capacity/mg•g^**−1**^**
Cyclohexane	22	13	62	85
Benzene	36	18	94	96
Isopropanol	38	25	84	103

Activated carbon is composed of carbon, hydrogen, oxygen, nitrogen and other elements and the content of carbon usually accounts for more than 90%. The skeleton structure of activated carbon is similar to amorphous carbon and it is mainly composed of graphite microcrystals in which 2~4 layers of single layer graphite sheets are stacked (Min et al., 2017; Raj et al., 2018). A large number of micropores are formed in the process of disorderly superposition of adjacent graphite microcrystals and the pore diameter of activated carbon micropore is between 0.7 and 2 nm. The activated carbon has large specific surface area due to its unique pore structure characteristics (Chmelik et al., 2010). The carbon atoms on the surface are easily oxidized with hydrogen and oxygen to form various functional groups due to covalent bond unsaturation in the preparation of activated carbon. Therefore, the polarity of activated carbon has significant effect on its adsorption properties (Bhatnagar et al., 2013; Prajapati et al., 2016).

Zeolite X has the function of screening molecules. Uniform pore size and honeycomb structure are the unique characteristics of zeolite X. The adsorbate can be captured when its molecular diameter is smaller than the zeolite X aperture (Zhou et al., 2014; Calero and Gómez-Álvarez, 2015; Brihi et al., 2018). The framework of the zeolite X carries negative charge because the silicon atoms are replaced by aluminum. The negative charge is compensated by strongly polar molecules outside the framework of the zeolite X. The greater polarity of the adsorbate molecules and the higher adsorption capacity of the zeolite X (Akosman and Kalender, 2010; Gorshunova et al., 2015; Wang et al., 2017). Adsorbate molecules are activated due to special electric field effects in the pore channels of zeolite X, which ensures that the zeolite X can identify the adsorbate molecules accurately and improve the adsorption performance of the zeolite X (Akosman and Kalender, 2010; Kim and Ahn, 2012). Brosillon et al. used zeolite to adsorb heptane and acetone respectively. The results showed that the adsorption capacity of zeolite to heptane was larger than that of acetone. The molecular polarity of heptane is stronger than acetone, which reflects the excellent selectivity of zeolite and it is closely related to the molecular polarity of VOCs (Brosillon et al., 2000).

## Conclusions

The comprehensive utilization of coal fly ash can not only develop circular economy, but also produce huge environmental and social benefits. At present, China's economy is developing at high speed and the demand for resources is also increasing as well. The refinement and high value-added comprehensive utilization of coal fly ash resources should be the prospect in the future and it is an important way to achieve sustainable development strategies in China. This study may bring a new and alternative approach for recycling of coal fly ash. By analyzing the chemical phase, microstructure and VOCs adsorption performance of zeolite X and activated carbon, it was found that:

- The study has verified that the preparation of zeolite X by the aluminum residue from coal fly ash is feasible. There is no need to add silicon or aluminum source during the synthesis process. It effectively enhances the comprehensive utilization efficiency of the coal fly ash and enriches the product categories.
- The prepared zeolite X is of high purity and crystallinity through the analysis of chemical phase. The crystalline form is mainly octahedral structure and the particle size distribution is basically uniform. The structure and microstructure specific of zeolite X are analyzed. The results show that the BET specific surface area is 990.3 m^2^/g and the pore diameter is 1.6 nm.
- Through the experiment of VOCs adsorption, it is found that the VOCs adsorption capacity exceeds the activated carbon. The adsorption capacity of cyclohexane on zeolite X is higher than that on benzene and isopropanol. The preparation of zeolite X by the aluminum residue from coal fly ash for the adsorption of VOCs conforms to the environmental protection concept of “treating waste with waste.” The energy-saving and environmental protection effect is obvious and it has strong application potential.

## Data Availability

All datasets generated for this study are included in the manuscript and/or the supplementary files.

## Author Contributions

All authors have contributed in various degrees to the analytical methods used, to the research concept, to the experiment design, to the acquisition of data, or analysis and interpretation of data, to draft the manuscript or to revise it critically for important intellectual content.

### Conflict of Interest Statement

The authors declare that the research was conducted in the absence of any commercial or financial relationships that could be construed as a potential conflict of interest.
